# Dissecting Highly Mutagenic Processing of Complex Clustered DNA Damage in Yeast *Saccharomyces cerevisiae*

**DOI:** 10.3390/cells10092309

**Published:** 2021-09-03

**Authors:** Stanislav G. Kozmin, Gregory Eot-Houllier, Anne Reynaud-Angelin, Didier Gasparutto, Evelyne Sage

**Affiliations:** 1 Institut Curie, PSL Research University Orsay, F-91405 Orsay, France; gregory.eot@univ-rennes1.fr (G.E.-H.); Anne.Reynaud@curie.fr (A.R.-A.); 2 Department of Molecular Genetics and Microbiology, Duke University School of Medicine, Durham, NC 27710, USA; 3 Institut de Génétique et Développement de Rennes, CNRS-UR1 UMR6290, Université Rennes-1, F-35043 Rennes, France; 4 CEA, CNRS IRIG/SyMMES-UMR5819, Université Grenoble Alpes, F-38054 Grenoble, France; didier.gasparutto@cea.fr; 5 CNRS UMR3347, INSERM U1021, Université Paris-Saclay, F-91405 Orsay, France

**Keywords:** clustered DNA damage, multiply damaged sites, non-DSB clustered DNA damage, oxidized base lesions, base excision repair, mutagenesis, mutagenic potential, ionizing radiation

## Abstract

Clusters of DNA damage, also called multiply damaged sites (MDS), are a signature of ionizing radiation exposure. They are defined as two or more lesions within one or two helix turns, which are created by the passage of a single radiation track. It has been shown that the clustering of DNA damage compromises their repair. Unresolved repair may lead to the formation of double-strand breaks (DSB) or the induction of mutation. We engineered three complex MDS, comprised of oxidatively damaged bases and a one-nucleotide (1 nt) gap (or not), in order to investigate the processing and the outcome of these MDS in yeast *Saccharomyces cerevisiae*. Such MDS could be caused by high linear energy transfer (LET) radiation. Using a whole-cell extract, deficient (or not) in base excision repair (BER), and a plasmid-based assay, we investigated in vitro excision/incision at the damaged bases and the mutations generated at MDS in wild-type, BER, and translesion synthesis-deficient cells. The processing of the studied MDS did not give rise to DSB (previously published). Our major finding is the extremely high mutation frequency that occurs at the MDS. The proposed processing of MDS is rather complex, and it largely depends on the nature and the distribution of the damaged bases relative to the 1 nt gap. Our results emphasize the deleterious consequences of MDS in eukaryotic cells.

## 1. Introduction

The spatial distribution and reparability of DNA damage are key parameters in the triggering of lethal or mutagenic events in cells. Clustered DNA damage, so-called Multiply Damaged Sites (MDS), are essentially induced by ionizing radiation; that is, when a single radiation track hits the DNA double helix associated with its water molecules, causing clusters of ionizations that lead to clusters of lesions, at the nanometer scale [1,2,3]. Clustered DNA damage is defined as two or more DNA lesions distributed on both strands within one or two helix turns, comprising a combination of oxidized bases, apurinic/apyrimidinic sites (AP sites), and single- or double-strand breaks (SSB and DSB, respectively). DSB, generated when two opposing SSB are produced in close vicinity, comprise a type of clustered DNA damage. Yet, non-DSB clustered DNA damage is at least three to eight times more frequently produced than DSB after exposure of cells to X-rays or gamma radiation [4,5,6]. The complexity of non-DSB clustered DNA damage increases with the linear energy transfer (LET) of radiation. Simulation studies have revealed that as many as 10 lesions per damaged site may be formed with high occurrence, depending on the LET [7,8,9]. For example, 4 MeV α-particles (LET 105 keV/μm) have been predicted to produce one five-lesion cluster, four two-lesion clusters, and eight isolated lesions in a DNA fragment [7]. The detection of clustered DNA damage has been reported in isolated DNA and in cells after exposure to ionizing radiation, including low-energy electrons, or even by H_2_O_2_-induced oxidative stress ([4,5,6,10,11,12,13,14], reviewed in [15]). High LET radiation, which induces clustered DNA damage at a much higher frequency than γ-rays, produces the exact same oxidatively damaged bases as γ-rays and with the same occurrence, only with a different distribution. The higher the LET, the more complex the clustered DNA damages [7]. However, due to a lack of high-resolution and -sensitivity analytical tools, the severity and complexity of DNA damage cannot be investigated. Complex DSB have recently been highly substantiated at the nanoscale level in cells by Lorat et al. [16], using Transmission Electron Microscopy. A combination of the use of high LET radiation, high-resolution microscopy, and spatiotemporal analysis of DNA damage patterns has allowed for the proposal that clustered DNA damage is responsible for chromatin rearrangement, chromosome breakage, persisting DNA damage, and genomic instability, highlighting its biological significance ([12,16,17,18,19,20,21,22,23] for review). Indeed, clustered DNA damage is considered as the most deleterious lesion induced by ionizing radiation.

Most of the available data on the processing and biological consequences of MDS have been assessed using a variety of synthetic models of clustered DNA damage, composed of base lesions, AP sites, and/or strand breaks. Studies mainly consider bi-stranded clusters, which often comprise one 8-oxo-7,8-dihydroguanine (oG) and one or two other lesions, such as anAP site, uracil, SSB, or oxidized/reduced base lesions (thymine glycol, dihydrothymine, 5-formyluracil), all possibly formed by ionizing radiation and repaired by BER. The effects of purine (oG) versus oxidatively damaged pyrimidine—mutagenic or not, replication-blocking or not—have been explored as well as the distance between lesions within the clusters. Biochemical investigations have established that the recognition and excision/incision of a lesion may be affected by the presence of opposite neighboring lesions, and that it largely depends on the composition, location, and distance between the lesions within the cluster. Thus, impaired repair within MDS may limit the formation of DSB. Such findings have been corroborated by in vivo studies using plasmid-based assays, mainly in *Escherichia coli*, but also in yeast and mammalian cells, and these have been extensively reviewed [21,24,25,26,27]. In short, initiation and the subsequent steps of BER of a lesion within MDS may be retarded, resulting in a persistent nearby lesion that causes mutation upon replication. Increased mutation frequency has been reported—in particular, at oG—in various combinations of non-DSB clustered damage [28,29,30,31,32]. Nonetheless, the fact that lesions at MDS can be partially processed or repaired at a reduced rate may favor the generation of DSB as repair intermediates. Indeed, MDS composed of bi-stranded U, AP sites, or Gap/AP sites are readily converted to DSB in bacteria and yeast [33,34,35,36,37], resulting in plasmid loss; while, in mammalian cells, DSB formed at opposite AP site analogues within MDS can be repaired by non-homologous end-joining and generate large deletions in the recovered plasmids [38,39,40]. In contrast, the processing of MDS containing oG on one strand and oG or another base lesion on the other strand, which results in a limited number of DSB, if any [37,41]. This demonstrates that the hierarchy in the lesion processing within MDS determines repair, DSB formation, mutability and ultimately, the outcome of clustered DNA damage [28,29,30,31,32,36,37].

We followed a slightly different approach, using more complex MDS comprising three to five lesions. We also used a plasmid-based assay in *E. coli* but did not apply a selection on the resulting transformants, which were directly analyzed by sequencing to detect mutation [36]. In addition, in contrast to most of the studies to date, which have employed DNA repair-deficient cells to observe substantial mutagenesis, we were able to detect mutations at MDS in a “normal” situation (i.e., in wild-type cells).

We aimed to elucidate the processing of lesions within complex MDS and their mutagenic potential. To this end, we built duplex oligonucleotides harboring complex MDS composed of three or four types of oxidative base damage (e.g., oG; 8-oxo-7,8-dihydroadenine, oA; 5-hydroxyuracil, hU; 5-formyluracil, fU) in combination (or not) with “dirty” SSB (one-nucleotide gap containing 5’-OH end) distributed on both strands within 17 bp. All the lesions, as single lesions, are readily formed by ionizing radiation. However, hU—a stable product of cytosine oxidation/deamination—is less common, and it is used here as a model of mutagenic oxidized pyrimidine. As mentioned above, our complex MDS could easily be formed by high LET radiation [7]. Using a biochemical approach with human and rodent cell extracts on the same constructs, we previously showed that the BER of an individual lesion within these complex MDS was impaired, largely depending on the nature and distribution of base damage and gap, and on the availability of repair proteins, thus limiting DSB formation [42,43]. The results suggest a hierarchy in the processing of the lesions within our complex MDS, which prevents DSB formation and should largely enhance mutagenesis. Then, we challenge our in vitro data in an in vivo situation. These MDS-containing DNA duplexes were inserted into yeast integrative and replicative plasmids, which served to transform yeast *Saccharomyces cerevisiae* and, using the relative transformation efficiency assay, we showed that DSB were not produced during repair at these MDS, in spite of the presence of a 1 nt gap [37]. We demonstrated that both in yeast [37] and *E. coli* [36], the kinetics of the initial repair event is a key parameter in the conversion (or not) of MDS into DSB. Here, to further dissect the repair at complex MDS, various MDS-containing duplexes (as well as singly damaged and undamaged control duplexes) were inserted into replicative plasmids, and the induced mutations at MDS were analyzed in WT and repair-deficient yeast cells. The in vitro excision/incision at base damage within MDS by the whole-cell yeast extracts was also investigated. The results demonstrated that complex MDS are hypermutagenic in normal, repair-proficient eucaryotic cells, emphasizing a pivotal role of clustered DNA damage in radiation-induced mutagenesis.

## 2. Materials and Methods

### 2.1. Oligonucleotides and Plasmids

**Figure 1:**
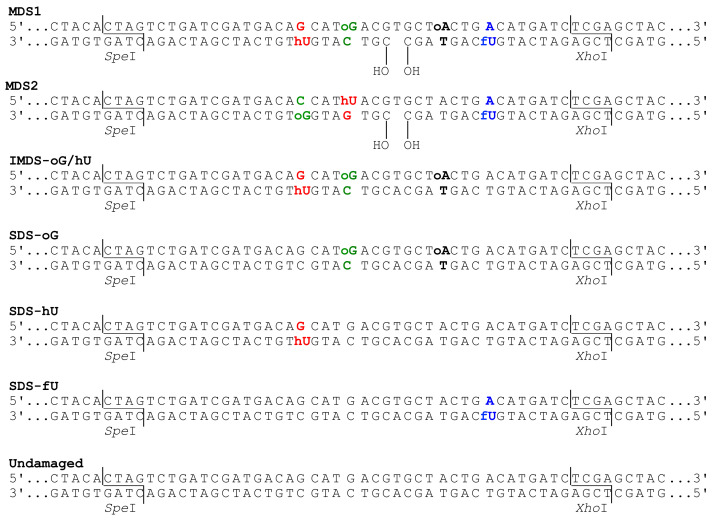
Duplexes carrying damaged sites used in this study. Oligonucleotides carried 8-oxoguanine (oG), 8-oxoadenine (oA), 5-hydroxyuracil (hU), and 5-formyluracil (fU). oG is located at 4 bp from hU in MDS1, MDS2, and IMDS-oG/hU. MDS1 and MDS2 carry a 1 nt gap terminated by 3′OH and 5′OH. All duplexes are 56 bp long and harbor *Spe*I and *Xho*I restriction sites at each terminus.

Unmodified oligonucleotides and oligonucleotides carrying 8-oxoguanine (oG), 8-oxoadenine (oA), 5-hydroxyuracil (hU), and 5-formyluracil (fU) were synthesized as previously described [42]. We built various singly and multiply damaged site (MDS) constructs of different orientation and complexity, as depicted in Figure 1. All duplexes harbored SpeI and XhoI restriction sites (Figure 1). A low-copy centromeric plasmid, pRS415 (Stratagene), was used for the introduction of oligonucleotides into yeast.

### 2.2. Yeast Strains and Media

All *Saccharomyces cerevisiae* strains used in this study were derived from strain FF18733 (MATa *his7-2 leu2-3,112 lys1-1 ura3-52 trp1-289*) (F. Fabre, CNRS-CEA, Fontenay-aux-Roses, France), and obtained from Dr. S. Boiteux (CNRS-CEA, Fontenay-aux-Roses, France). The following set of FF18733 derivatives was used: FF181134 (*rev3*Δ::*URA3*), BG300 (*rad14*Δ::*kan*MX6), CD182 (*ntg1*Δ::*URA3 ntg2*Δ::*TRP1*), BG310 (*ntg1*Δ::*URA3 ntg2*Δ::*TRP1 rad14*Δ::*kan*MX6) [44], BG3 (*apn1*::*URA3*, *apn2*::*kan*MX6) [45], and DGD39 (BER^-^) (*ung1*Δ *ntg1*Δ *ntg2*Δ::*kan*MX6 *ogg1*Δ::*URA3 mag1*Δ::*hph*MX4) [37]. Complete YEPD medium and synthetic complete medium SC without leucine (SC-LEU) were used [46]. For the selection of ampicillin-resistant *Escherichia coli* colonies, Luria broth medium was supplemented with 100 μg/mL of ampicillin (LB+Ap) [47].

### 2.3. Yeast Transformation and Analysis of Transformants

Plasmid pRS415 was double-digested with *Spe*I and *Xho*I. The linearized vectors were purified by agarose gel electrophoresis using a Geneclean Turbo Kit (Q-BIOgene). A total of 100 pmol of single-stranded oligonucleotides were annealed in 1× restriction buffer #2 (New England Biolabs) to form the appropriate duplexes, which were digested simultaneously with *Spe*I and *Xho*I for 1 h at 37 °C. Then, the oligonucleotides were purified using a QIAquick Nucleotide Removal Kit (QIAGEN). Concentrations of vectors and of oligonucleotides were quantified by UV absorbance. A total of 200 ng of linearized pRS415 were ligated with 10 ng of each *Spe*I/*Xho*I-digested duplex using T4 DNA ligase (New England Biolabs) in supplied buffer for 16 hr at 16 °C. The whole amount of each ligation mixture was used to transform yeast, according to the LiAc procedure [46]. Yeast cells were plated on SC-LEU plates, and Leu^+^ clones were colony-purified on SC-LEU plates. One colony from each parental clone was grown in liquid SC-LEU medium, and total DNA was isolated using the miniprep procedure [46]. Then, DNA samples were electroporated into *E. coli* XL1-Blue strain (Stratagene), and Ap^r^ cells carrying plasmids were selected on LB+Ap plates. Plasmid DNA from a single colony from each transformation was isolated and used for sequencing. Sequencing was performed using universal T3 primer (5′-ATTAACCCTCACTAAAG-3′) (Stratagene) at the GENOME Express Company (Meylan, France).

### 2.4. Preparation of Yeast Cell Extract

The cell-free extract was prepared from 200 mL of wild-type yeast exponential culture (approximately 10^7^ cells/mL). After washing with ice-cold water, yeast cells were pelleted and resuspended in 500 µL of lysis buffer (25 mM Tris-HCl, pH 7.5; 5 mM EDTA; 250 mM NaCl; and 0.6 mM PMSF). Then, 2 mL of glass beads (size 425–600 μm) were added, and the cell suspension was vortexed 5 times over 1 min (between vortexing cycles, the extract was kept on ice). Then, another 500 µL of lysis buffer were added, and the suspension was vortexed 3 more times over 1 min. After removing the glass beads by centrifugation, the cell extract was ultra-centrifuged for 30 min at 30,000 rpm at 4 °C in a Beckman Coulter Optima MAX Ultracentrifuge, using a TLA-55 rotor. Then, glycerol was added (20% vol. final) and total protein concentration in the extract was measured using Bradford reagent (BIORAD). The cell extract was aliquoted and stored at −20 °C.

### 2.5. Cleavage of the Lesion-Containing Duplexes by Yeast Cell Extract

Oligonucleotides were 5′-^32^P-end-labeled as previously described [42]. Unincorporated nucleotides were removed using ProbeQuant G-50 Micro Columns (GE Healthcare). After phenol/chloroform extraction, the radiolabeled oligonucleotides were hybridized with an equimolar quantity of radiolabeled complementary strand or 1.75 excess of the non-radiolabeled complementary strand in hybridization buffer (140 mM NaCl; 10 mM Tris-HCl, pH 8; 1 mM EDTA, pH 8), by heating for 5 min at 95 °C and slow cooling to room temperature. The hybridization efficiency was verified by the migration of DNA samples on a native 12% polyacrylamide gel (19:1; acrylamide/bisacrylamide *w*/*w*; 100 mM Tris-borate; 1 mM EDTA, pH 8).

The cleavage assay mixtures (14 µL final volume) contained 100 fmoles of radiolabeled double-stranded oligonucleotides and yeast cell extract (10 μg of proteins) in the incubation buffer (20 mM Tris-HCl, pH 7.6; 140 mM NaCl; 4 mM EDTA, pH 8; 8% glycerol). The reactions were performed at 37 °C for 0.5 to 4 h and stopped by the addition of stop-buffer (0.5% SDS and 50 mM EDTA, pH 8). Proteinase K (Eurobio) was added at 0.8 mg/mL, which was followed by incubation for 1 h at 37 °C. After phenol/chloroform extraction and ethanol precipitation, samples were loaded on a 12% denaturing polyacrylamide gel (19:1; acrylamide/bis-acrylamide *w*/*w*; 7 M urea; 100 mM Tris-borate; 1 mM EDTA, pH 8). The reaction products were visualized and quantified using the Molecular Dynamics Storm 820 PhosphorImager and ImageQuant 5.2 software (Molecular Dynamics). The cleavage efficiency is expressed as the percentage of the amount of cleaved molecules to the total amount of (i.e., cleaved plus uncleaved) molecules.

### 2.6. Repair Synthesis Assay on MDS1 by Yeast Whole-Cell Extract

The reaction mixture (30 uL) contained 300 fmol of MDS1 duplex, labeled at the 5′-end of the strand, carrying fU, 20 mM Tris-HCl (pH 7.6), 75 mM NaCl, 5 mM MgCl_2_, 1.5 mM EDTA, 4 mM ATP, 2 mM NAD, 40 mM phosphocreatine, 0.05 mg/mL creatine phosphokinase, 0.25 mg/mL BSA, 6% glycerol, and 50 μg yeast whole-cell extracts, plus 20 μM of each dNTP or not. The repair reaction was performed at 37 °C for 3 h, and it was stopped by adding SDS/EDTA stop buffer. Then, the reaction mixture was incubated at 37 °C for 1 h in the presence of proteinase K (20 μg), and DNA was further purified by phenol/chloroform extraction and ethanol precipitation. Reaction products were separated by electrophoresis on 18% polyacrylamide, 7 M urea gel.

### 2.7. Statistical Analysis

A two-tailed Fisher’s exact test was applied to compare mutation spectra occurring in various constructs and in various strains. Comparison of the oligonucleotide cleavage efficiency in vitro was performed using an independent two-tailed Student’s test for equal-variance samples. Equality of variances was tested using an F-test.

## 3. Results

To gain insight into the processing of complex MDS, we built duplex oligonucleotides harboring three to four oxidized base lesions which, as single lesions, are possibly excised by different DNA *N*-glycosylases with different kinetics. We also explored the impact of a “dirty” SSB and the location of base lesions regarding the SSB on the outcome of the MDS. Figure 1 depicts all the MDS and Singly Damaged Sites (SDS-oG, SDS-hU, SDS-fU) used as controls. MDS1 comprises oG and oA at 8 nt on the upper strand, and hU and fU at 8 nt from a 1 nt gap terminated by 5′- and 3′-OH on the lower strand. The residue hU is paired with G and fU is paired with A, in order to simulate the oxidation of C and T, respectively, by ionizing radiation in DNA. IMDS-oG/hU is similar to MDS1 but without fU and the 1 nt gap. In MDS2, oG and hU are in an inverted position, relative to the 1 nt gap, and oA is missing. Despite the presence of oA in the SDS-oG oligonucleotide, we rather refer to it as a single oG-control, because of complete lack of oA-induced mutations in all tested contexts and the very unlikely BER of the oA•T pair in yeast (see below). The damaged or undamaged DNA duplexes were inserted into the replicative plasmid pRS415 and used to transform yeast cells, and the processed plasmids were recovered from transformants. The relative transformation efficiencies of these constructs have previously been analyzed in wild-type yeast cells [37]. It revealed a total absence of DSB formation during the processing of the lesions, for any of these constructs. This encouraged us to sequence the transformants and analyze the associated mutations.

### 3.1. Elevated Mutagenesis Is Targeted at Damage within MDS

Plasmids carrying MDS1, MDS2, IMDS-oG/hU, or singly damaged site SDS-oG, SDS-hU, SDS-fU, and the undamaged DNA duplex were first used to transform wild-type yeast cells.

**Figure 2:**
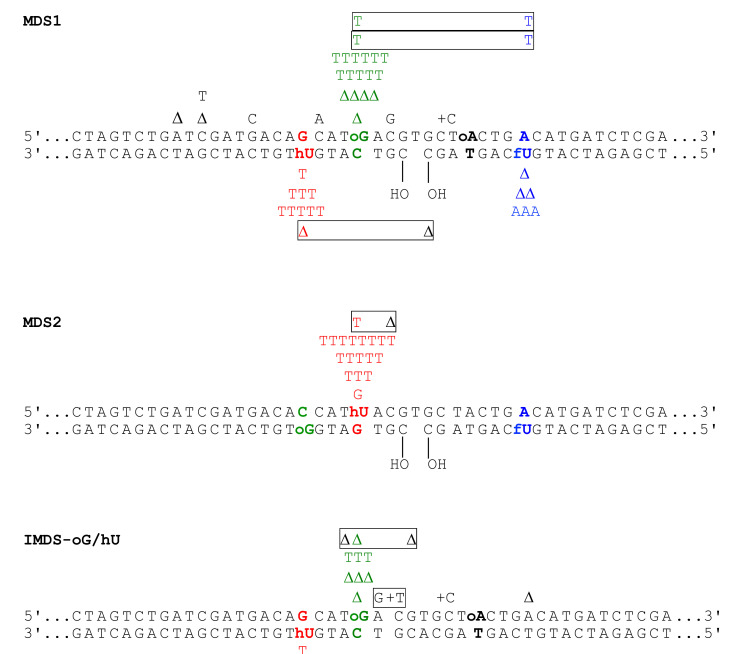
Mutation spectra induced at complex MDS in the WT yeast strain. Positions of the lesions and targeted mutations are bolded and color marked. Red indicates the hU•G pair, green indicates the oG•C pair, black indicates the oA•T pair, and blue indicates the fU•A pair. Mutations in black are untargeted. Double mutations are boxed. Frequencies of non-targeted mutations in IMDS-oG/hU, MDS1, and MDS2 are 10%, 9.5%, and 4%, respectively.

A general and detailed characterization of the mutations induced at MDS1 is presented in Table S1 as well as in Figure 2 and Figure S3. It is revealed that a vast majority of mutations within MDS1 occur at base damage, although non-targeted mutations were also observed: in 460 MDS1 samples sequenced, 188 (85%) mutations were targeted to sites of lesions, and 34 (15%) mutations occurred at other positions (see Table S1, last column). Similar results were obtained with the other MDS and SDS studied (Figure 2 and Figure S1). The frequencies of non-targeted mutation ranged between 3 and 10%, which is a level of mutation similar to that we have previously observed in *E. coli* [36]. It is possible that the generation of some of these mutations—at least, those adjacent to a lesion—are directly related to the presence of the lesion, as has been reported in similar assays [48]. Thus, we sequenced samples of the pRS415 plasmid carrying unmodified oligonucleotide after replication in wild-type yeast. Mutations were found in 19% of samples (15 out of 79 sequenced), and they were equally distributed as 1 nt deletions or base substitutions dispersed among the oligonucleotide sequence (Figure S2a). Notably, only one mutation (a deletion) was located at the site corresponding to a lesion (fU) in MDS1 and MDS2. We suspect that due to the size of the oligonucleotides, which was rather elevated (up to 56 mers), the methods for purification and purity analysis might be not sensitive enough to detect a few percent of 1 nt oligonucleotides, undesirable bases, or sugar modifications generated during the chemical synthesis that are mutagenic. Indeed, the mutation frequency and spectrum at an undamaged DNA duplex of the same size replicated in *E. coli* was very similar to those at the undamaged DNA duplex replicated in yeast in the present work (16% vs. 19%, deletion, base substitution and double mutation) [36]. Similarly, an unexpected level of mutations within the oligonucleotide sequence was observed when vectors containing undamaged synthetic oligonucleotides were replicated in *E. coli* [49,50]. The analysis of the sites of mutation in an undamaged duplex versus in MDS1 in Figure S2b clearly demonstrates that a vast majority of the mutations within MDS1 were targeted to the sites of lesions, with high significance.

Then, we focused on the targeted mutations, detailed the mutation types, and calculated the mutation frequencies (MFs) observed at oG, hU, and fU for each complex MDS, after which we compared them to those at singly damaged sites (Table 1A–C). It appears that the types of targeted mutation at all three MDS did not differ from that at SDS. Mutations at oG were oG•C to T•A transversions, as expected [48,51,52,53], but 1 nt deletions were also frequent at MDS and at SDS-oG (half of the mutations at oG in SDS-oG, 63% in IMDS-oG/hU, and 28% in MDS1). Mutations targeted at hU were almost all hU•G to T•A transitions, which was the exclusive mutation type observed at hU in *E. coli* [36,49]. Indeed, hU reads as T by replicative DNA polymerases, thus being either repaired or mutagenic. fU induced fU•A to A•T transversions and 1 nt deletions. Such types of mutations have also been recovered at fU in *E. coli* and in mammalian cells [50,54]. Notably, no mutation was recovered at oA paired with T in MDS1, IMDS-oG/hU, or SDS-oG. No other data on the mutagenicity of oA in yeast have been published to date. Similar to our findings, oA within a single-stranded vector was not mutagenic in *E. coli* [55]. However, in 3T3 mouse fibroblasts, oA induced A->G and A->C mutations in the 5′-CC**oA**AG-3′ sequence context [56]. On the other hand, in COS-7 Simian kidney cells, oA was only slightly mutagenic in the 5′-CT**oA**GC-3′ sequence context, and it was not mutagenic within the 5′-TG**oA**CC-3′ sequence [57]. In vitro, some polymerases (e.g., *Taq* or *E. coli* PolI) exclusively introduce dTTP opposite oA [58], whereas other polymerases (such as human Polβ and Polη) can frequently incorporate dGTP opposite oA [59]. Unfortunately, no data on oA being bypassed by yeast DNA polymerases are available. No cleavage of DNA oligomers containing oA paired with T was detected in human nuclear and mitochondrial extracts [60]. On the other hand, significant cleavage (approximately 20%) of an oA•T-containing oligonucleotide has been observed in a mouse cell extract due to the action of thymine-DNA glycosylase, TDG [61]. In *E. coli* cell extract, a very weak cleavage (<1%) of the oA•T-containing template was due to the action of a mismatch-specific uracil-DNA glycosylase, Mug [61]. Neither TDG nor Mug homologs are present in *S. cerevisiae*, and yOgg1 glycosylase is unable to recognize oA•T substrates [62]. Thus, we believe that the absence of oA-targeted mutations in yeast most likely reflects its limited miscoding capacity rather than its hyper-efficient repair. Taken together, the types of mutations at base damage clustered within complex MDS are the same as at isolated lesions.

**Figure 3:**
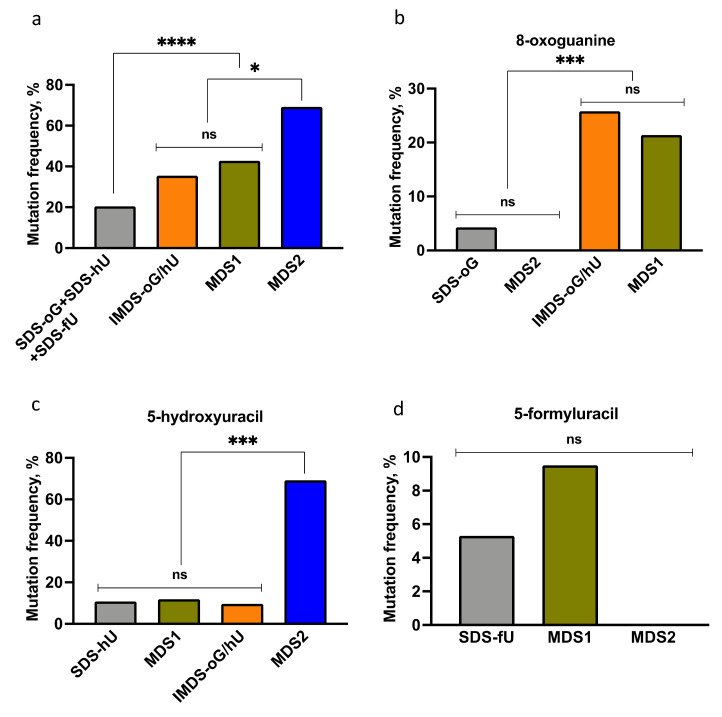
Mutation frequencies at complex MDS, as compared to singly damaged sites, in the WT yeast strain. (**a**) Total mutation frequencies at IMDS-oG/hU, MDS1, and MDS2 in comparison with the sum of mutation frequencies at singly damaged sites (sum of SDS vs. IMDS-oG/hU, MDS1, or MDS2, *p* < 10^−4^; IMDS-oG/hU vs. MDS1, *p* = 0.53; IMDS-oG/hU vs. MDS2, *p* = 0.017; MDS1 vs. MDS2, *p* = 0.025). (**b**) Targeted mutation frequencies at 8-oxoguanine (SDS-oG vs. IMDS-oG/hU, *p* = 0.0016; SDS-oG vs. MDS1, *p* = 0.0004). (**c**) Targeted mutation frequencies at 5-hydroxyuracil (SDS-hU vs. MDS2, *p* < 10^−4^). (**d**). Targeted mutation frequencies at 5-formyluracil. Statistical analysis was performed using Fisher’s exact test. *p*-values abbreviated as ****: *p* < 10^−4^; ***: 10^−4^ < *p* < 10^−3^; *: 10^−2^ < *p* < 0.05; ns: non-significant (*p* ≥ 0.05).

The next step was to compare the frequencies of the targeted mutations at IMDS-oG/hU, MDS1, and MDS2 to that at SDS. The mutation frequencies (MFs) are summarized in Figure 3. First, Figure 3a shows that the total MF at each of the three studied MDS was significantly and largely higher than that of the sum of MFs at SDS. This demonstrates that MDS are highly mutagenic. It is noteworthy that the MF at IMDS-oG/hU, carrying three damaged bases, was not statistically different from the MF at MDS1, carrying four damaged bases and a 1 nt gap. In addition, the MF at MDS2 was significantly higher than that at MDS1, although these two MDS mainly differed by an inverted position of hU and oG.

Secondly, the MF at each damaged base, oG, hU, and fU within the three MDS were compared to the MF at SDS-oG, SDS-hU, and SDS-fU (Figure 3b–d). The residue oG was highly mutated in MDS1 and IMDS-oG/hU (five to six-fold above SDS-oG), while it was not in MDS2 when located on the opposite strand at 8 nt from the 1 nt gap (Figure 2). The absence of mutation at oG in MDS2 was likely due to the low number of sequenced clones (26 instead of 93 for SDS-oG; Table 1A), but it may also be due to the reduced survival of the oG-containing strand. MF at hU within MDS1 and IMDS-oG/hU was the same as for SDS-hU, while it is more than six-fold higher in MDS2 (Figure 3c). This implies that hU exhibits a similar level of repair in the context of MDS1 and IMDS-oG/hU as an isolated lesion, while it is not (or poorly) repaired in the context of MDS2 when located at 4 nt opposite the 1 nt gap. Moreover, the MF at hU in MDS2 was above 50%, suggesting that some portion of mutations opposite hU might be introduced during long-patch (≥5 nt) repair DNA synthesis at SSB (1 nt gap) on the opposite strand. Alternatively, the higher-than-expected MF at hU might be due to preferable replication of the strand carrying hU and reduced survival of the opposite DNA strand. The MFs at fU in MDS1, MDS2, and SDS-fU did not significantly differ (Figure 3d). The absence of mutation at fU in MDS2 was likely due to the low number of sequenced clones (26 instead of 94 for SDS-fU; Table 1C), but it may be also due to the reduced survival of the fU-containing strand. All the MFs at MDS1 and IMDS-oG/hU (Table 1A,B, Figure 3a–c) were not significantly different and, accordingly, their mutation spectra are similar, beside the fact that fU was not present in IMDS-oG/hU (Figure 1). This indicates that the presence of the 1 nt gap within MDS1 did not increase the mutagenic potential in this configuration. Moreover, hU, which was not more mutated in MDS1 than in SDS-hU, is highly mutagenic in the context of MDS2. Altogether, these data reveal that the distribution of damaged bases is a key parameter, which may be more important than the presence of a break (1 nt gap), per se, to induce mutations at clustered lesions. In addition, mutations may occur at damaged bases on both strands (Table S1, Figure 2 and Figure S3). This reveals the complexity of the repair process at MDS.

### 3.2. Processing of Lesions within MDS

The analysis of mutations induced at MDS in wild-type yeast cells already brings some information on the capacity of the lesions to be repaired within the MDS. To further dissect the processing of the lesions within MDS, we examined the mutation induced at MDS1 in various base excision repair (BER)-deficient yeast strains and in *rev3* cells deficient in lesion bypass DNA polymerase zeta (Polζ). We also studied the excision/incision at the most mutated base damage—oG and hU—with the three MDS duplex by whole-cell yeast extracts, deficient (or not) in BER. Our cleavage assay was set to reveal the first steps of BER; for example, excision of the damage base and incision of the DNA backbone at the abasic site generated by the base excision [37,42,43]. It would not be appropriate to detect excision by nucleotide excision repair (NER), although the DNA duplexes are 56 bp long.

**Figure 4:**
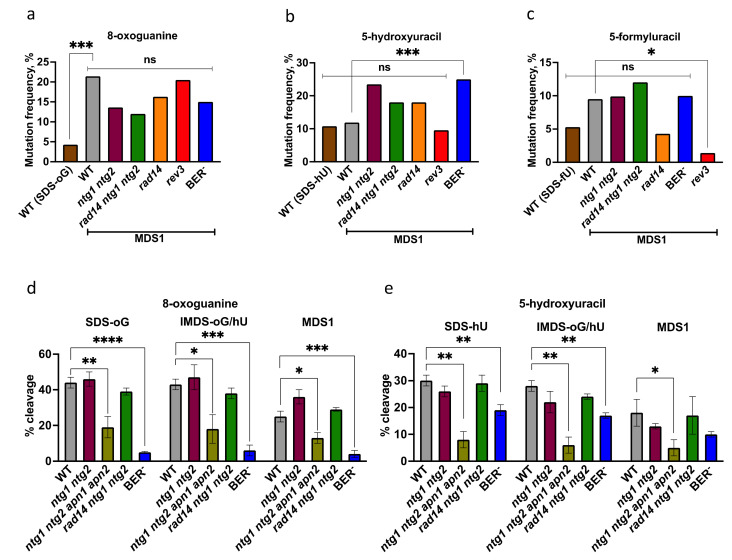
Mutation frequencies at MDS1 in DNA repair-deficient yeast strains and cleavage at damaged sites by yeast whole-cell extract. (**a**) The mutation frequencies observed at 8-oxoguanine in MDS1 in the various strains do not significantly differ (Fisher’s exact test). (**b**) The mutation frequencies observed at 5-hydroxyuracil in MDS1 in the repair-deficient strains do not differ from WT, except in BER^-^ strain (BER^-^ vs. WT, Fisher’s exact test, *p* = 0.042). (**c**) The mutation frequencies observed at 5-formyluracil in MDS1 in the repair-deficient strains do not differ significantly from WT, except in the *rev3* strain (Fisher’s exact test, *p* = 0.039). (**d**) The extent of cleavage at oG within IMDS-oG/hU, MDS1 by the whole-cell extracts from the various yeast strains, after 2 h incubation, is compared to that in SDS-oG. In WT, MDS1 vs. SDS-oG, *p* < 0.01; in *rad14 ntg1 ntg2*, MDS1 vs. SDS-oG, *p* < 0.01 (*t*-test). (**e**) The extent of cleavage at hU within IMDS-oG/hU, MDS1 by the whole-cell extracts from the various yeast strains, after 2 h incubation, is compared to that in SDS-hU. In WT, MDS1 vs. SDS-hU, *p* < 0.05; in *ntg1 ntg2*, MDS1 vs. SDS-hU, *p* < 0.01; in BER^-^, MDS1 vs. SDS-hU, *p* < 0.01 (*t*-test). For technical reasons, the cleavage at fU in MDS1 was not studied. Fisher’s exact test or *t*-test *p*-values are abbreviated as follows: ****: *p* < 10^−4^; ***: 10^−4^ < *p* < 10^−3^; **: 10^−3^ < *p* < 10^−2^; *: 10^−2^ < *p* < 0.05; ns: non-significant (*p* ≥ 0.05).

We first explored the roles of BER proteins and the translesion synthesis Polζ in the mutagenesis at MDS1, using mutated yeast strains. The overall mutation types and frequencies in all tested strains resembled that in the wild-type strain, as revealed in Table S1, and lesion-targeted mutagenesis occurred with similar specificities to that found in the wild-type strain (Table 2A–C). This is reflected in Figure S3, which gathers the mutation spectra of MDS1 in the various strains. Table 2A–C and Figure 4a–c reveal that two observations were statistically significant (*p* < 0.05): (i) the two-fold increased mutagenesis at hU in the *ung1 ntg1 ntg2 ogg1 mag1* (BER^-^) strain; and (ii) the reduced mutagenesis at fU (in particular, the absence of fU•A to A•T transversions) in the *rev3* strain. The former suggests that hU within the MDS was partly repaired and BER was involved. More likely, Ntg1, Ntg2, and Ung1 proteins were required to repair hU as, in vitro, yeast Ntg1 and Ntg2 and *E. coli* uracil-DNA-glycosylase are able to excise hU from irradiated DNA [63,64]. The latter observation suggests that Polζ is involved in the mutagenic bypass of fU. Notably, it was demonstrated that the *rev3* mutation suppressed the spontaneous mutator phenotype of the yeast *ntg1 ntg2 apn1* strain, which implies a role of Polζ in the bypass of oxidized pyrimidines [65]. As the DNA conformation at MDS1 is likely disturbed due to the presence of the 1 nt gap and the multiple damaged bases within 17 nt, we could expect that NER might be involved in the processing of MDS1. A synergism between BER (mediated by Ntg1 and Ntg2) and NER (Rad14 and Rad1) has been observed in yeast [44,65]. However, our data clearly indicated the absence of a role of Rad14/NER in the processing of MDS1.

Next, we examine the in vitro cleavage efficiency at the MDS by the repair-deficient yeast cell extracts with regard to the mutation frequencies at the MDS in these strains.

Cleavage at oG in SDS-oG, IMDS-oG/hU, and MDS1 by yeast extracts.

The cleavage at oG by WT yeast extract was impaired in MDS1 (25%), in comparison with SDS-oG (44%) (*p* < 0.01, *t*-test), while it was not in IMDS-oG/hU (43%) (Figure 4d). A retarded repair of oG in MDS1 was also observed, using purified yOgg1 (data not shown). The inhibition of oG repair at MDS1 was likely due to the presence of the 1 nt gap near oG, which is absent in SDS-oG and IMDS-oG/hU. The oG-excision inhibition at MDS1 was even more pronounced with human and rodent cell extracts [42,43]. As expected, the cleavage at oG was abolished in the extract from BER^-^ cells lacking all known yeast DNA *N*-glycosylases, including Ogg1. The absence of Ntg1 and Ntg2, which are not involved in oG excision, seemed to slightly improve the cleavage at oG in MDS1, possibly by reducing the repair proteins competition for binding at MDS, although it was not statistically significant. Interestingly, the cleavage at oG, either isolated or within the MDS, was significantly reduced (about two-fold, relatively to WT) in the absence of Ntg1, Ntg2, Apn1, and Apn2, indicating an important role of AP-endonuclease in the processing of Ogg1-generated abasic sites (Figure 4d). It has been reported that in human cells, the major AP endonuclease Ape1 stimulates hOgg1 activity by accelerating the turnover of hOgg1, thus bypassing its AP-lyase activity [66,67,68]. It has not been reported in yeast, but it seems that Apn1/2 also remove yOgg1 from the AP site after oG excision and cleave the AP site. Moreover, we observed that the cleavage product by *ntg1 ntg2 apn1 apn2* extract migrated slightly differently to that of all the other cell extracts, suggesting an incision on the 3′ of the oG position in the absence of Apn1/2 and on the 5′ side in the presence of the Apn1/2 (Figure S4a). Finally, inactivation of the nucleotide excision repair (NER) factor Rad14, which is involved in the recognition of damage, had no role in the cleavage at oG in SDS-oG, IMDS-oG/hU, or MDS1 (Figure 4d). Altogether, Ogg1 and Apn1/2 are required for the BER of oG within the studied substrates—at least, in vitro—and their efficiency was reduced in MDS1 (likely due to the presence of the 1 nt gap). Moreover, while the MF at oG was about five times higher within MDS1 than in SDS-oG, it was not further increased in the strain lacking Ogg1 (BER^-^) (Figure 4a). Altogether, these data are consistent with a delayed repair of oG within MDS1, leading to replication of the oG-containing strand before oG repair.

Cleavage at hU in SDS-hU, IMDS-oG/hU, and MDS1 by yeast extracts.

For all tested strains, the cleavage efficiency at hU in IMDS-oG/hU and in SDS-hU did not significantly differ, whereas, in MDS1, it was slightly lower than in SDS-hU for three strains (*t*-test *p* < 0.01 for BER^-^ and *ntg1 ntg2*; *p* < 0.05 for WT) (Figure 4e). The weak and non-significant decrease in the cleavage of all hU-containing substrates in *ntg1 ntg2* backgrounds suggests another pathway than Ntg1/Ntg2 in the repair of hU. In the absence of Ntg1 Ntg2, it is probable that the monofunctional DNA-*N*-glycosylase Ung1 excises hU, and that the AP site is incised by Apn1/2, as deduced from the significantly lower extent of hU cleavage in BER^-^ extract lacking Ntg1, Ntg2, and Ung1, in comparison with WT. Interestingly, Apn1/2 played a substantial role in BER of hU in all studied substrates, as demonstrated by the very weak hU cleavage in the *ntg1 ntg2 apn1 apn2* extract (Figure 4e). This extract cannot incise the AP site left by Ung1. As we observed at oG, in such an extract, the cleavage product at hU migrates slower than those in the presence of Apn1/2 (Figure S4b). Thus, Apn1/2 may also be involved in the recycling of Ntg1/Ntg2 and incision at AP site after Ntg1/Ntg2 action, similarly to what we observed with yOgg1 (see above), although the effect of Ape1 on hNth—the homologue of the Ntg—is not as clear as for hOgg1 [69]. On the other hand, we (and others) have shown that human AP endonuclease Ape1 is able to incise DNA at hU [42,70], which may also be the case for Apn1/2. This could explain the remaining cleavage in the BER^-^ extract. The similar hU-cleavage efficiency observed in *rad14 ntg1 ntg2* and *ntg1 ntg2* cell extracts indicates that Rad14—and, thus, NER—does not seem to play a role in hU excision, in any context (Figure 4e). Noteworthy, in the PAGE analysis, we did not observe any DNA fragment that could correspond to the excision of either oG or hU within the MDS duplex by NER. This does not exclude that NER proteins cannot bind DNA at MDS; in particular, Rad14 and Rad4–Rad23 are involved in damage recognition. Collectively, the in vitro data suggest a critical role of Apn1/2 for cleavage at hU, as well as Ntg1, Ntg2, and Ung1, and a slightly less efficient excision/incision at hU within MDS1 as compared to SDS-hU. However, the MF at hU in SDS-hU and MDS1 in WT cells is the same, suggesting that the processing of hU in MDS1 is effective (see Figure 4b). Furthermore, the MF at hU in MDS1 is two times higher in BER^-^ cells than in WT (see Figure 4b), demonstrating that in vivo, Ntg1/Ntg2/Ung1 are the major DNA-*N*-glycosylases involved in the repair of hU within MDS1. In contrast, NER does not play a significant role in the repair of hU, and Polζ is not involved in the mutagenic bypass of hU. Collectively, the results show a good capacity of hU to be repaired in vivo—likely by Ntg1/Ntg2.

We have shown that fU was poorly excised by human whole-cell extract; yet, we observed that the excision/incision at fU within MDS1 was impaired and about two times lower than at SDS-fU [42]. We also found that the cleavage at fU within MDS1 by yeast whole-cell extract was very poor and could not be investigated further (Figure S5). Excision of 5-fU from double-stranded DNA has been demonstrated in vitro with *S. cerevisiae* Ntg1 and Ntg2 proteins [71]. Figure 4c shows that fU is mutagenic in yeast but at a similar level as oG in SDS-oG. MF at fU in MDS1 was not increased in cells lacking Ntg1 and Ntg2. Moreover, the two-fold difference in the MFs at fU within SDS-fU and MDS1 was not statistically significant, suggesting an unaffected processing of fU within MDS1. NER deficiency did not exhibit any effect on fU-induced mutagenesis within MDS1. In contrast, Polζ seems to be involved in the mutagenic bypass of fU.

Table S1 and Figure S3 reveal that targeted double mutations occurred in all the tested strains and involved the combinations oG and hU, oG and fU, and hU and fU. They occurred with a frequency of 1–6%, except in the BER^-^ (*ung1 ntg1 ntg2 ogg1 mag1*) strain, in which the frequency was increased to 14% (the difference with wild-type strain was statistically significant; Fisher’s exact test, *p* = 0.025). The increase in the frequency of double mutations in the BER^-^ strain correlated with enhanced mutagenesis at 5-hU in this strain, as most of the double mutations (eight out of 11) involved 5-hU. Targeted double mutations are not likely to be induced during repair by BER.

**Figure 5:**
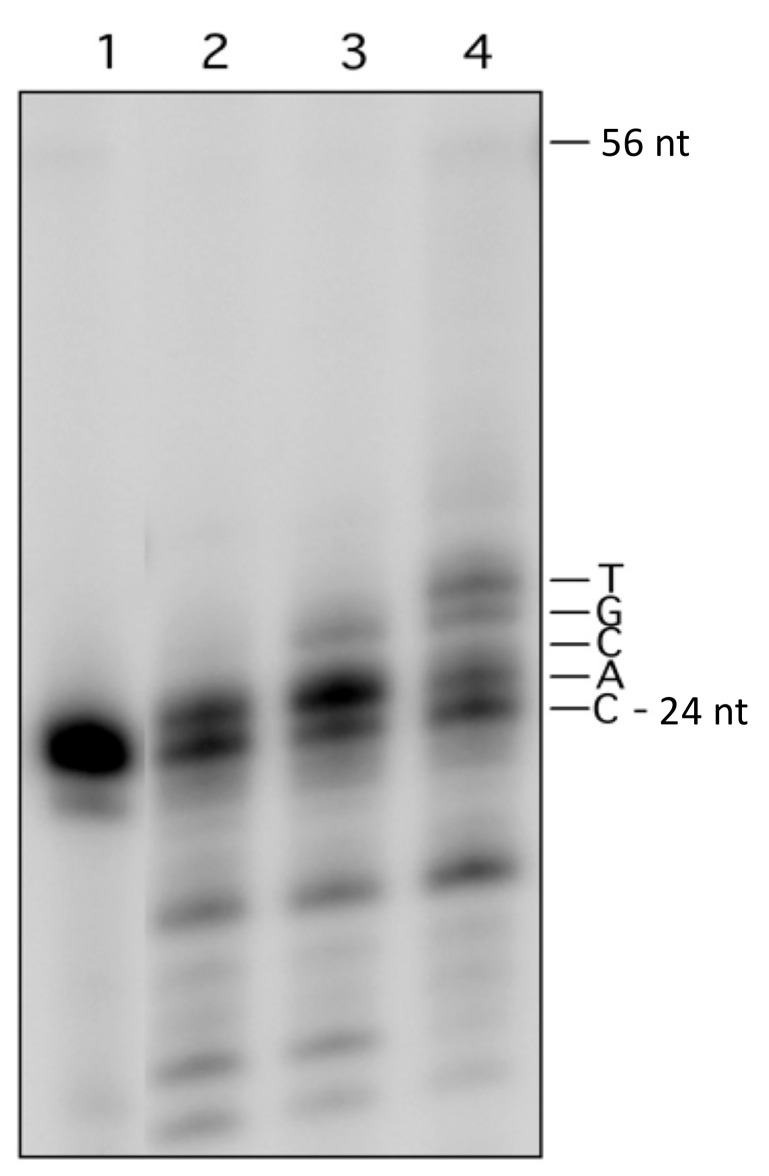
Repair synthesis at MDS1 by yeast whole-cell extract. MDS1 duplex labeled at the 5′ end of the 24 mer strand carrying fU were incubated with (lanes 2–4) or without (lane 1) yeast whole-cell extract in the absence of additional dNTP, other than those likely present in the extract (lane 2), in the presence of 20 μM dATP (lane 3) or 20 μM of 4 dNTP (lane 4). A full-length DNA product that could possibly represent synthesis and ligation at the 1 nt gap or a full copy of the oG-containing strand was not observed. Note that bands below the 24 mer in lanes 2–4 are degradation products, due to exonucleases in the extract, which were activated in the presence of MgCl_2_ in the reaction mixture.

Indeed, MDS1 and MDS2 carry a 1 nt gap bordered by 5′-OH and 3′-OH termini (Figure 1), which cannot be simply repaired by a 1 nt insertion followed by ligation. More likely, the repair of this lesion occurs through a ‘long-patch’ pathway, which requires the incorporation of a few nucleotides by Polδ/ε, strand displacement, and removal of the 5’-flap by Rad27 [72,73]. To further support this process, we performed in vitro repair synthesis by yeast whole-cell extract and showed that dATP was readily incorporated on the 3′OH terminus of the gap, opposite the T (Figure 5). Furthermore, in the presence of the four dNTPs, the four intense bands seen on the electrophoregram indicate an incorporation of 4 nt and the occurrence of strand displacement; nearly no further synthesis was observed after the incorporation of the 4 nt. Altogether, the results demonstrated that the damaged bases nearby did not prevent the initiation of repair synthesis at the gap and may have favored strand displacement. It also showed that the polymerase involved stops or made a strong pause at the nucleotide before oG. This is fully consistent with in vitro data, indicating three to six-fold reduced bypass of oG (relatively to undamaged G) by yeast Polδ [74]. In addition, Budworth et al. [75], using an oligonucleotide duplex containing oG located one nucleotide 3′ of an SSB with a 3′OH end, showed that oG constitutes a strong block for human Polδ, and that polymerization was nearly completely blocked after addition of the first nucleotide. We also examined repair synthesis on MDS1 and similar MDS with human Polδ/ε, in the presence of PCNA for Polδ. The results clearly indicated that the 1 nt gap of MDS1 is an entry site for both eucaryotic polymerases, and that both Polδ/ε perform strand displacement, make a strong pause at oG (stopping 1 nt before oG or incorporating a nucleotide opposite oG), and are able to bypass oG (data not shown). In addition, the extent of the pause site by Polδ depends on the location of oG relative to the 1 nt gap. A long-patch repair of the 1 nt gap could prevent BER of damaged base oG or hU in MDS1 or MDS2 and generate mutations. Notably, in the yeast *S. cerevisiae*, there is no short patch BER, as Polβ is missing.

## 4. Discussion

To gain insight into the processing and the mutagenic potential of clustered DNA damage, most studies have been conducted in *E. coli*, and a few have been conducted in mammalian cells. A specific assay has been carried out in the yeast *S. cerevisiae*, in order to investigate the DSB formation at clustered DNA damage and the role of homologous recombination in its repair [76]. We found it interesting to focus on three closely related MDS, more complex than those studied, and examine their repairability and mutability, taking advantage of the availability of BER-deficient *S. cerevisiae* mutants. The fact that mutations were detected by the direct sequencing of transformants (after amplification in *E. coli*), without any filter, allowed for observation of all of the possible mutational events; this was not the case for most of the published studies. Thus, we should gain a better knowledge of the mutability of MDS and provide comprehensive models for the processing of complex MDS in eukaryotic cells.

The present study investigated the mutation types and frequencies induced at three MDS—namely, MDS1, MDS2, and IMDS-oG/hU, which were comprised of three or four oxidized bases (oG, hU, fU, oA), a 1 nt gap (or not), in two different configurations—in yeast *S. cerevisiae* proficient or not in BER or TLS. We have previously examined most of the repair events that could occur at these MDS, using human and rodent cell extracts [42,43]. We also have carried out an in vivo/vitro study in yeast *S. cerevisiae* cells, investigating the formation of DSB at these MDS [37]. The presented results are first discussed in light of our previous set of data to propose a processing for each of the three MDS.

We undoubtedly eliminated the possibility of the simultaneous repair (excision/incision) of opposed damaged bases within the MDS or excision/incision of oG (or oA) in MDS1 or hU in MDS2, in the presence of the 1 nt gap in yeast [37]. Indeed, the comparable transformation efficiency of the plasmids harboring the MDS to that of controls allowed us to exclude DSB formation as a repair intermediate, which was supported by the same transformation efficiencies in wild-type cells and in *rad52* cells deficient in homologous recombination required for DSB repair [37]. This result allows us to suppose that mutations would occur. A major finding of the present work is that complex clustered DNA damage is highly mutagenic in wild-type yeast *S. cerevisiae*, with the vast majority of mutations targeted at lesion sites within the MDS. In addition, double mutations targeted at base damage also occur in MDS1 and IMDS-oG/hU. The elevated MF values observed in wild-type cells were much greater than those observed in most previous reports (see [25] and the references therein). Although, in vitro, a relative capacity of excision/incision at oG and hU in MDS remained detectable (Figure 4d,e), the elevated MFs at oG in MDS1 and IMDS-oG/hU, and at hU in MDS2, revealed a dramatic repair inhibition of these damaged bases in their respective context in cells. Notably, the lack of increase in MF at oG in MDS1 in BER^-^ cells lacking Ogg1 (Figure 4a) indicated a total absence of oG repair in that case. Another substantial finding was that NER did not seem to be involved in the repair of our MDS, and that only BER is. This may be surprising, as the three to four damaged bases and the 1 nt gap (supposed to introduce a bend in the phosphodiester backbone, [77]) are likely to induce some distortion of the DNA double helix within the MDS. It has been reported that bacterial NER UvrABC endonuclease is able to efficiently incise DNA at a tandem lesion composed of thymine glycol and 2′-deoxyribonolactone or tetrahydrofuran abasic site [78]. It has also been shown that the NER of bulky guanine adducts is totally abolished when an AP site is located on the opposite strand [79]. To the best of our knowledge, NER has not been involved in the processing of non-DSB clustered damage. Another important aspect of the results is the dissimilarity in the mutation spectra of the two related MDS1 and MDS2, differing by only an inverted position of oG and hU. This indicates that different mechanisms may operate to lead to the elevated MFs at MDS1 and MDS2. The replication of unrepaired oG or hU in our MDS was not sufficient to explain these observations—in particular, for hU in MDS2 (MF = 69%). Altogether, our results indicate a highly mutagenic processing of MDS.

**Figure 6:**
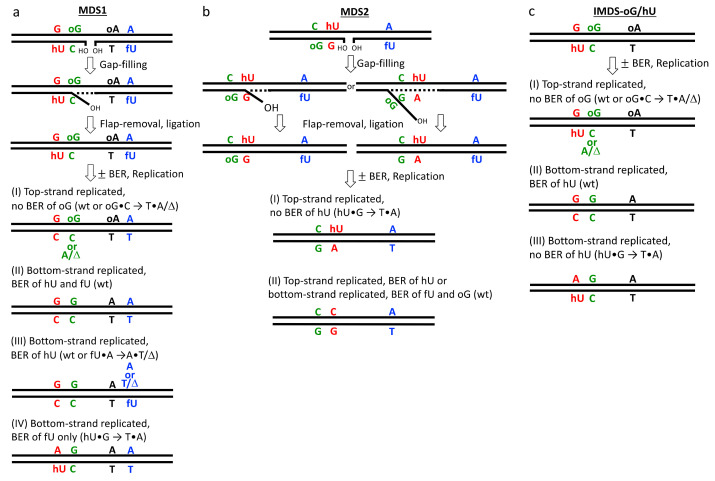
Models for in vivo processing of MDS1 (**a**), MDS2 (**b**), or IMDS-oG/hU (**c**), leading (or not) to mutations. Most probable events are shown, based on mutation spectra and in vitro data (see Section 4).

Processing at MDS1 (Figure 6a). The mutagenesis data show that only the MF at oG was significantly and highly increased in MDS1 (compared to MF in SDS-oG), and only hU was subject to repair in vivo, whereas oG was not (Figure 4a–c). The in vitro activity of Ogg1 in the extract at MDS1 was significantly impaired, in comparison to SDS-oG and IMDS-oG/hU (Figure 4d), due to the presence of the 1 nt gap nearby on the opposite strand [42,43]. The in vivo absence of (or limited) oG repair within MDS1 prevents DSB formation [37]. Similar results have been thoroughly reported in the literature for other simpler constructs, and increased mutation at oG has been observed as a consequence (reviewed in [21,24,25]). While the extent of cleavage at hU by mammalian cell extract was as high in MDS1 as in SDS-hU, it was slightly lower in the yeast extract. Regardless, in vivo, hU within MDS1 exhibited a good capacity to be excised by Ntg1/Ntg2 and Ung1 (Figure 4b, BER^-^ strain), emphasizing that oG situated at 4 nt on the opposite strand did not affect hU excision (see also IMDS-oG/hU on Figure 4d). fU paired with A did not seem to be efficiently excised by Ntg1/Ntg2, as there was no statistically significant increase in MF at fU in repair-deficient cells, relative to wild-type cells, as well as in MDS1, relative to SDS-fU (Figure 4c). oA paired to T is likely not repaired by BER and is not mutagenic in yeast. We have previously shown that the rejoining of the 1 nt gap by human cell extract was delayed in MDS1, in comparison to that at a single gap [42]. Using a human cell extract and purified repair proteins, we have also shown that the 1 nt gap was repaired mostly by short-patch repair (1 nt), but that strand displacement may also occur, allowing for a few more nucleotides to be incorporated. Indeed, the delayed repair of SSB by DNA ligase III/XRCC1 has been observed when present in clustered DNA damage [80]. In vivo, there may be competition between hU and the gap for binding of the repair proteins, DNA *N*-glycosylases, and SSB-repair proteins. However, the similarity in the MFs and spectra in MDS1 and IMDS-oG/hU suggested that the 1 nt gap was quickly filled through ≤4 nt patch repair DNA synthesis (with following flap removal and ligation), and that this event occurs before hU excision; otherwise, we should expect an increase in oG-induced mutations in MDS1 in comparison with IMDS-oG/hU. The reason why DNA synthesis initiated at SSB in MDS1 essentially terminated before oG is likely due to the poor ability of yeast replicative DNA polymerases to bypass oG. Indeed, the in vitro bypass efficiency of oG by yeast DNA polymerase δ has been found to be only 16–31% of the value for the undamaged (G-containing) template [74]. This is also supported by the results in Figure 5, showing that DNA synthesis—likely involving Polδ/ε—was stopped 1 nt before oG, with very low bypass. We propose that this repair sequence is the major one happening at MDS1. Then, in MDS1 (and in IMDS-oG/hU as well), the SSB generated by excision/incision at hU fully inhibits the repair of oG. In fact, the lack of increase in oG-targeted mutations at MDS1 in the strain missing *ogg1* (BER^-^) supports the idea that in both cases, the repair of oG is nearly 100% inhibited by nearby lesions (hU in IMDS-oG/hU and hU+1 nt gap in MDS1), and what we see is the maximal MF at oG that can be possibly observed. In such processing, replication takes place before oG can be repaired, causing mutation at oG, which is likely by Polδ/ε. The MF at hU and fU were the same as in controls, SDS-hU and SDS-fU; mutations at unrepaired hU and fU likely also occurred during replication.

However, at MDS1, some mutations occasionally arose during gap filling; this was the case for double-targeted mutations (Table S1). The existence of double-targeted mutations oG+hU (in BER^-^, *rad14*, and *rev3* strains) indicated that 5–8 nt gap-repair DNA synthesis opposite oG occurred. Furthermore, it happened before hU could be excised. Then, the mutations at oG and hU were fixed, during replication of the hU/fU-carrying strand. In some other rare cases, double mutations were observed at oG+fU (in wt, BER^-^, and *rad14 ntg1 ntg2* strains). In those cases, the first event was also ≥5 nt gap-repair DNA synthesis, which was followed with hU elimination either by Fen1 within a flap or by BER; A was incorporated opposite oG during repair synthesis and mutation was fixed during replication of the fU-carrying strand, when mutation opposite fU occurred. Another example was given in the absence of BER, with the significant increase in double mutations at hU+fU. The gap filling occurred first, likely by ≤8 nt patch DNA synthesis, and mutations at hU and fU occurred during replication. These events were favored by the absence of BER.

Processing at MDS2 (Figure 6b). Mutations were exclusively observed at hU, with an extremely high frequency (69%), suggesting a very poor repair of hU, if any. Meanwhile, the cleavage at hU by yeast extract is still more rapid than at oG [37]. Moreover, the rate (extent and kinetics) of cleavage at hU in MDS2 by mammalian cell extract was similar to that in MDS1 and SDS-hU [42,43]. This could mean that in vivo, the first event is the gap filling associated with a near-complete lack of hU repair and, therefore, part of the mutations at hU occur during gap-filling synthesis when the repair patch is ≥5 nt. Possibly, in contrast to MDS1, where gap filling usually terminates at oG (see above), DNA synthesis at MDS2 can freely overpass hU, similarly to what was demonstrated in vitro with yeast DNA polymerases δ and ε and uracil-containing template [81]. The displaced strand may be long enough (>8 nt) to carry oG. Then, it is removed by Fen1 and followed by ligation. Thus, the MF at hU equal to 50% may be due to replication of the unrepaired hU-containing strand, whereas the additional 19% may be generated during gap-filling synthesis (assuming 100% mutagenicity of hU—originating from C—when paired with G). Alternatively, the above 50% MF at hU may suggest loss of the strand carrying oG and fU, which could be due to an unsealed gap or replication fork collapse caused by the binding of repair proteins that attempt to repair in the MDS. The absence of mutation at oG and fU in MDS2 could be due to strand loss; however, it could also be due to the low number of rescued plasmids sequenced, as this reduction of MFs at oG and fU in MDS2, relative to corresponding SDS, was statistically insignificant (Figure 3b,d). Then, the lack of similar strand loss in MDS1 (manifested as equal MF at oG in MDS1 and IMDS-oG/hU) can be explained by the inability of Ogg1 to bind to the site, which is due to the more rapid initiation of BER at hU on the opposite strand [42,43], as discussed above. Strand loss during the processing of clustered DNA damage has been proposed, although not demonstrated in *E. coli* [31,82]. We also proposed strand loss when we observed above 85% of mutation at hU in *E. coli* for another type of construct, implying a partly repaired uracil (SSB) and oG nearby on the same strand and hU next to U in the opposite strand [36]. Thus, despite the very similar structures of MDS1 and MDS2, the processing of these clusters occurs by different mechanisms, which are dictated by the nature of oxidative DNA lesions and their location within the cluster, leading to the dramatic differences in mutation frequencies and spectra observed in these MDS.

Processing at IMDS-oG/hU (Figure 6c). The kinetics of excision/incision at hU in MDS1, MDS2, and IMDS-oG/hU by yeast and mammalian cell extracts are faster than that at oG [37,42,43]. For example, in MDS2, regardless of the presence of the 1 nt gap nearby on the opposite strand, the initial cleavage at hU (<1 h) by yeast cell extract was at least two times faster than that at oG [37]. In IMDS-oG/hU, DSB are formed neither in vitro by yeast extract nor in yeast cells [37]. In fact, reports from the literature have converged on a faster and more efficient repair of oxidatively damaged pyrimidine than oG [83,84]. Figure 4e shows very similar cleavage efficiency at hU in IMDS-oG/hU and SDS-hU, whatever the strain. In addition, the MF values at hU in IMDS-oG/hU, SDS-hU, and MDS1 were exactly the same (Figure 3c), while the MF at oG was elevated in IMDS-oG/hU, as in MDS1 (Figure 3b). We can conclude that in IMDS-oG/hU, the initial event is the excision of hU, which is as efficient as in SDS-hU, and which dramatically impedes oG repair. Furthermore, the mutations at oG and hU occur during replication.

Outcome of the processing of non-DSB clustered damage/multiply damaged sites. From the processing described above for MDS1 and MDS2, the first event is the same: the repair of the 1 nt gap (Figure 6a,b). However, depending on the position of oG and hU, relative to the gap, the outcome is very different. The reason for this is that the length of the repair synthesis differs, which is likely due to the different efficiencies in the bypass of oG and hU by Polδ/ε. We saw that if the repair synthesis mainly stops at oG in MDS1, in some rare cases, it may pass oG. The bypass efficiency depends on the nature of the damaged base and, for example, it should be totally abolished at thymine glycol, which is not mutagenic but is a strong block to replicative polymerases [85]. It also depends on the location of the damaged base, relative to the SSB or the gap, as mentioned in Section 3. It may also depend on the stability of the double helix at the site; for example, the presence of another damaged base in close proximity may favor bypass. Then, the occurrence of mutations depends on the length of the repair patch, meeting or not a mutagenic lesion. However, in a number of cases, an SSB or a 1 nt gap may be repaired by the insertion of a single nucleotide followed by ligation (eventually a phosphatase or polynucleotide kinase cleans up the ends, if necessary). Therefore, an SSB or 1 nt gap in a non-DSB clustered DNA damage may not always be of utmost importance; it retards the BER of most damaged bases, but it may be repaired with some delay and without generating mutations. This is what occurs, most of the time, at MDS1, which (after gap filling) is processed similar to IMDS-oG/hU (Figure 6a,c). Then, BER excises the oxidatively damaged pyrimidine, and the induced SSB inhibits the repair of oG, leading to mutation upon replication. At that stage, BER has both beneficial and detrimental effects [86]; for example, the BER of hU at MDS1 or IMDS-oG/hU protects from mutagenesis at hU but strongly increases mutagenesis at oG. However, if a non-mutagenic oxidatively damaged pyrimidine would be present at similar position in MDS, instead of hU, only the detrimental aspect would remain. Long-patch repair synthesis at MDS has various consequences: (1) It may inhibit the repair of a mutagenic damaged base on the template strand (e.g., hU in MDS2); (2) it may eliminate damage base by strand displacement and removal of the lesion-carrying flap by an appropriate endonuclease (e.g., oG in MDS2); and (3) it may introduce mutations opposite a lesion in a template strand (e.g., at hU in MDS2 or oG in MDS1). On the other hand, replication fork collapse at clustered DNA damage may generate DSB and requires homologous recombination to overcome the blockage. Importantly, as the absence of simultaneous repair of bi-stranded damaged bases abolishes or limits DSB formation, strand displacement or strand loss protects from mutation by eliminating base damage. Through the example of our three MDS, we can conclude that the processing at MDS is rather complex and depends on many parameters, including the nature of the damaged bases, the presence of an SSB/gap, and the spacing between lesions within a cluster. The outcome of non-DSB clustered damage, comprising oxidatively damage bases plus SSB (or not) is essentially point mutation—simple or multiple—at a high frequency. It is worth noting that the processing of non-DSB clustered damage comprising AP site(s), in addition to oxidatively damage bases and/or SSB, generates DSB and point mutations ([36] and reviews [21,24,25]); this is not discussed here.

## 5. Conclusions

The present in vitro/vivo work, using synthetic multiply damaged sites, allowed for a better understanding of the early steps arising at MDS. We showed that the processing at this type of DNA damage is not unique but rather complex and may have different outcomes. Interestingly, a damaged base may be mutagenic in one context but not in another. Key parameters determine the sequential repair events at MDS: (i) The presence of an SSB within the cluster prevents/retards the repair of proximal DNA lesions, and the SSB is the first to be repaired; (ii) the capacity of repair DNA polymerases to bypass DNA lesions and their fidelity will determine the delay of the subsequent repair process and its mutagenic potential, respectively; and (iii) the intrinsic affinity and properties of DNA glycosylases direct the sequence of remaining DNA lesions processing. Thus, all of these steps will slow or abolish the repair of the last lesions composing the MDS, favoring their replication-induced mutagenesis. Our results demonstrated that MDS comprised of oxidatively damaged purines, pyrimidines, and SSB/gap are highly mutagenic, and that their processing leads mainly to point mutations (and, possibly, to strand loss). All of this highlights the deleterious consequences of radiation-induced clustered DNA damage.

## Figures and Tables

**Table 1:** Mutation type and frequency at oG, hU, and fU in SDS or complex MDS in WT strain.

**(A). Mutations at oG.**				
**Sequence at oG•C Site**	**SDS-oG**	**IMDS-oG/hU**	**MDS1**	**MDS2**
oG•C -> G•C (“WT”)	89	23	63	26
oG•C -> T•A	2	3	13	0
oG•C -> Δ	2	5	5	0
Total mutations	4	8	18	0
Total clones	93	31	84	26
Mutation frequency	4%	26%	21%	<4%
**(B). Mutations at hU.**				
**Sequence at hU•G Site**	**SDS-hU**	**IMDS-oG/hU**	**MDS1**	**MDS2**
hU•G -> C•G (“WT”)	83	28	74	8
hU•G -> T•A	9	3	9	17
hU•G -> Δ	1	0	1	0
hU•G -> G•C	0	0	0	1
Total mutations	10	3	10	18
Total clones	93	31	84	26
Mutation frequency	11%	10%	12%	69%
**(C). Mutations at fU.**				
**Sequence at fU•A Site**	**SDS-fU**		**MDS1**	**MDS2**
fU•A -> T•A (“WT”)	89		76	26
fU•A -> A•T	1		5	0
fU•A -> Δ	4		3	0
Total mutations	5		8	0
Total clones	94		84	26
Mutation frequency	5%		10%	<4%

**Table 2:** Mutation type and frequency at oG, hU, and fU in MDS1 in DNA repair-deficient strains.

**(A). Mutations at oG.**							
	**MDS1**						**SDS-oG**
**Sequence at oG•C Site**	**WT**	**BER^-^**	***ntg1 ntg2***	***rad14***	***rad14 ntg1 ntg2***	***rev3***	**WT**
oG•C -> G•C (“WT”)	66	68	70	77	44	58	89
oG•C -> T•A	13	4	6	8	4	9	2
oG•C -> Δ	5	7	5	7	2	6	2
oG•C -> C•G	0	1	0	0	0	0	0
Total mutations	18	12	11	15	6	15	4
Total clones	84	80	81	92	50	73	93
Mutation frequency	21%	15%	14%	16%	12%	21%	4%
**(B). Mutations at hU.**							
	**MDS1**						**SDS-hU**
**Sequence at hU•G Site**	**WT**	**BER^-^**	***ntg1 ntg2***	***rad14***	***rad14 ntg1 ntg2***	***rev3***	**WT**
hU•G -> C•G (“WT”)	74	60	62	81	41	66	83
hU•G -> T•A	9	20	18	7	9	5	9
hU•G -> Δ	1	0	0	4	0	2	1
hU•G -> G•C	0	0	1	0	0	0	0
Total mutations	10	20	19	11	9	7	10
Total clones	84	80	81	92	50	73	93
Mutation frequency	12%	25%	24%	18%	18%	10%	11%
**(C). Mutations at fU.**							
	**MDS1**						**SDS-fU**
**Sequence at fU•A Site**	**WT**	**BER^-^**	***ntg1 ntg2***	***rad14***	***rad14 ntg1 ntg2***	***rev3***	**WT**
fU•A -> T•A (“WT”)	76	72	73	88	44	72	89
fU•A -> A•T	5	6	6	3	4	0	1
fU•A -> Δ	3	2	2	1	1	1	4
fU•A -> G•C	0	0	0	0	1	0	0
Total mutations	8	8	8	4	6	1	5
Total clones	84	80	81	92	50	73	94
Mutation frequency	10%	10%	10%	4%	12%	1%	5%

## Data Availability

The data presented in this study are available on request from the corresponding author.

## Supplementary Materials

The following are available online at https://www.mdpi.com/article/10.3390/cells10092309/s1, Figure S1: Mutation spectra of singly damaged oligonucleotides in WT strain; Figure S2: a. Mutation spectrum of undamaged oligonucleotide in WT. b. Vast majority of mutations in MDS1 are targeted to sites of lesion; Figure S3: Mutation spectra of the MDS1 in various strains; Figure S4: Cleavage at oG (a) or hU (b) within MDS1 by whole-cell extracts from various yeast strains; Figure S5: Cleavage at oG, hU, and fU within MDS1 by whole-cell extracts from wild-type strain; Table S1: General characterization of the mutations in the MDS1-containing oligonucleotide within pRS415 plasmid after its replication in yeast.
