# Supplementary material for: Dissecting Highly Mutagenic Processing of Complex Clustered DNA Damage in Yeast Saccharomyces cerevisiae
Source: Cells. 2021 Sep 3;10(9):2309. doi: 10.3390/cells10092309 (PMC8471780; doi:10.3390/cells10092309)
Supplement: Supplementary file 1 [file cells-10-02309-s001.zip › cells-1315696-supplementary.pdf]

Supplementary Information

Table S1. General characterization of the mutations in the MDS1-containing oligonucleotide within pRS415 plasmid after its replication in yeast.

Sequence present at MDS:	Strain (relevant genotype)						Σ
	**wt** No (%)	***ung1 ntg1 ntg2 ogg1 mag1*** No (%)	***ntg1 ntg2*** No (%)	***rad14 ntg1 ntg2*** No (%)	***rad14*** No (%)	***rev3*** No (%)	No (%)
wild type[a]	43 (51)	46 (58)	38 (47)	28 (56)	59 (64)	48 (66)	262
							(57)
Single mutations:	38 (45)	23 (29)	42 (52)	19 (38)	28 (30)	24 (33)	174
targeted	31 (37)	20 (25)	36 (44)	15 (30)	24 (26)[c]	21 (29)	(38)
non-targeted	7 (8)	3 (4)	6 (7)	4 (8)	4 (4)	3 (4)	147
Double mutations:	3 (4)	11 (14) [b]	1 (1)	3 (6)	5 (5)	1 (1)	(32)
**targeted + targeted**	2 (2)	9 (11) [b]	1 (1)	3 (6)	1 (1)	1 (1)	27 (6)
oG + hU	-	2 (3)	-	-	1 (1)[d]	1 (1)	24 (5)
oG + fU	2 (2)	1 (1)	1 (1)	1 (2)	-	-	17 (4)
hU + fU	-	6 (8)	-	2 (4)	-	-	4 (1)
**targeted + non-targeted**	1 (1)	2 (3)	-	-	4 (4)	-	5 (1)
oG + non-targeted	-	2 (3)	-	-	1 (1)	-	8 (2)
hU + non-targeted	1 (1)	-	-	-	2 (2)	-	7 (2)
fU + non-targeted	-	-	-	-	1 (1)[e]	-	3 (0.7)
							3 (0.7)
							1 (0.2)
Total samples	84	80	81	50	92	73	460

[a] - sequence identical to the undamaged oligonucleotide (see Fig. 1).
[b] - significantly higher than observed in the wild-type strain (Fisher's Exact Test, $p < 0.05$).
[c] - one hU-targeted mutation counted as "single" was a 4-nt deletion involving hU.
[d] - also contains third mutation, a 1-nt deletion adjacent to hU.
[e] - also contains third mutation, a 1-nt deletion adjacent to fU.

```
SDS-oG
                            ΔΔ
                            T
                   C        T  Δ   +C
5'...CTAGTCTGATCGATGACAGCAToG ACGTGCToACTGACATGATCTCGA...3'
3'...GATCAGACTAGCTACTGTCGTAC TGCACGA T GACTGTACTAGAGCT...5'


SDS-hU

5'...CTAGTCTGATCGATGACAG CATGACGTGCTACTGACATGATCTCGA...3'
3'...GATCAGACTAGCTACTGThUGTACTGCACGATGACTGTACTAGAGCT...5'
                       T   ACA  T          +A
                      TTT
                     TTTTT
                    [A A]


SDS-fU
5'...CTAGTCTGATCGATGACAGCATGACGTGCTACTG A CATGATCTCGA...3'
3'...GATCAGACTAGCTACTGTCGTACTGCACGATGACfUGTACTAGAGCT...5'
                        T     Δ  A     A
                                      ΔΔΔΔ
```

Figure S1. Mutation spectra of singly damaged oligonucleotides in WT strain.

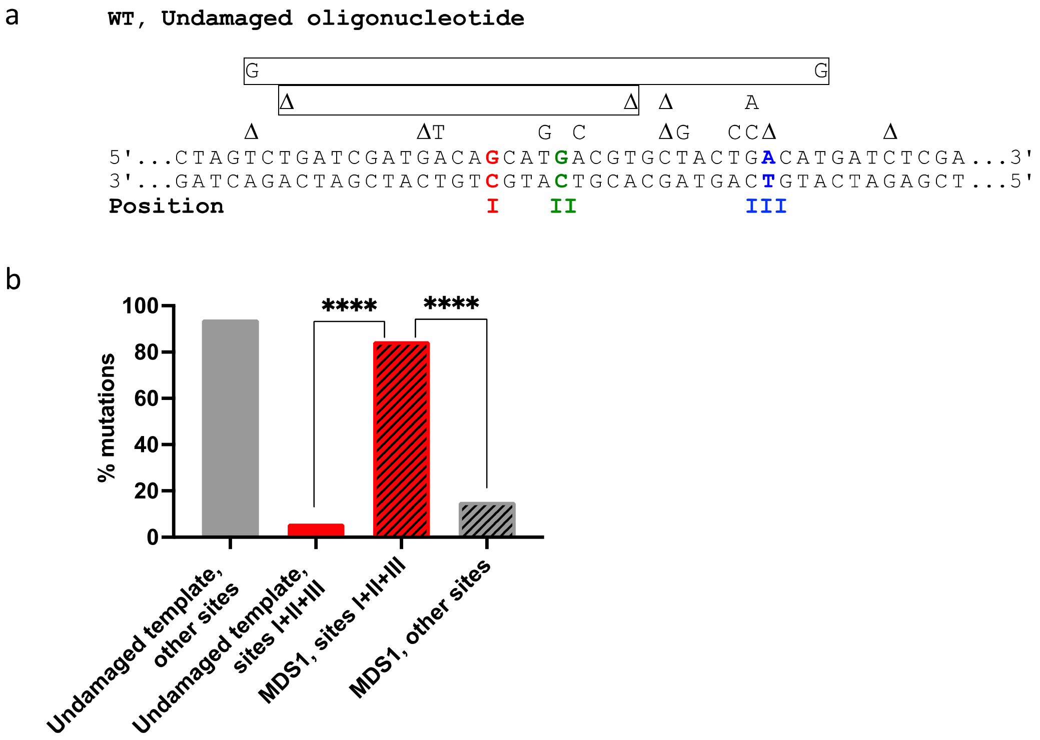

Figure S2. **a.** Mutation spectrum of undamaged oligonucleotide in WT. **b.** Vast majority of mutations in MDS1 are targeted to sites of lesion. For MDS1, data for all strains were combined: 460 samples were sequenced; 222 mutations were recovered; 188 (85%) mutations occurred at sites I+II+III and 34 (15%) mutations occurred at other positions. If mutations occurred randomly, the expected distribution of mutations within the 46-bp MDS1 oligonucleotide would be: $222 \times 3/46 = 15$ at sites I+II+III and $222 - 15 = 207$ at other positions, which is dramatically different from observed distribution (Fisher's exact test, $p < 10^{-4}$). For undamaged template, 79 samples were sequenced; 1 (7%) mutation occurred at site III, 14 (93%) mutations occurred at sites other than I, II or III. The difference between MDS1 and undamaged oligonucleotide mutation spectra is statistically significant (Fisher's exact test, $p < 10^{-4}$).

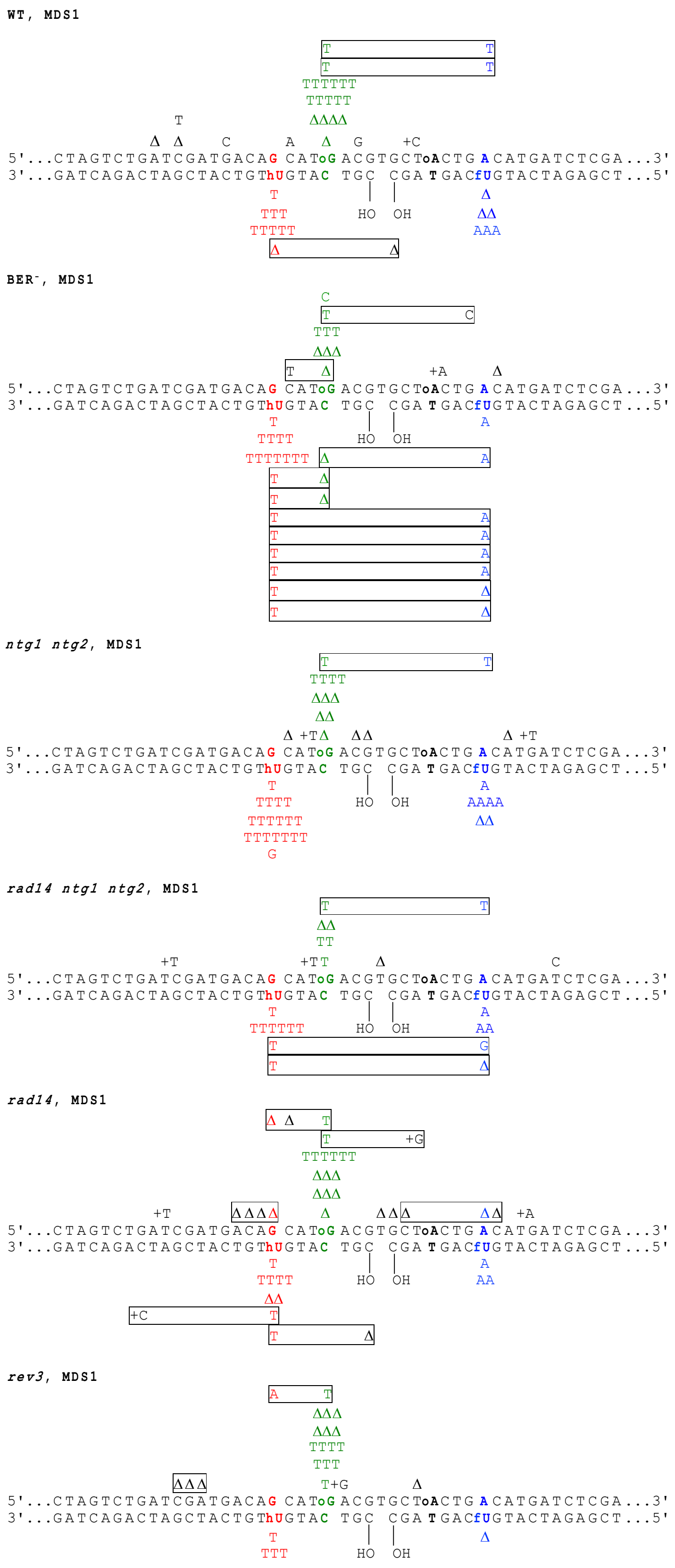

Figure S3. Mutation spectra of the MDS1 in various strains.

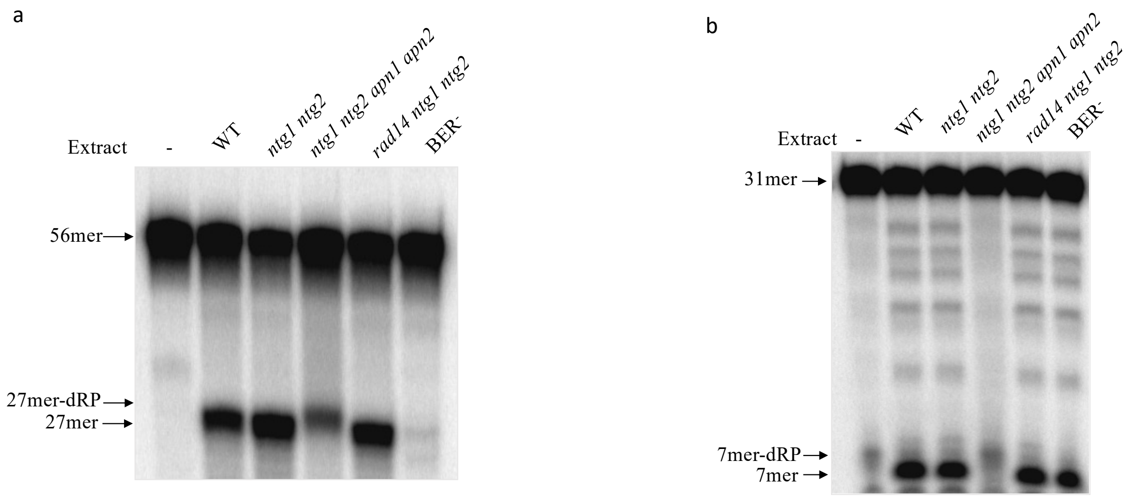

Figure S4. Cleavage at oG (a) or hU (b) within MDS1 by the whole cell extracts from the various yeast strains.

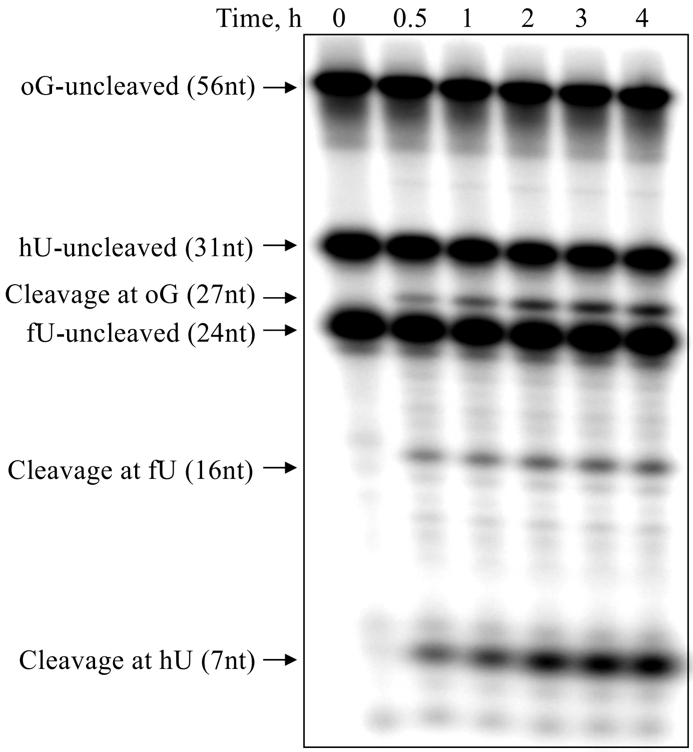

Figure S5. Cleavage at oG, hU, and fU within MDS1 by the whole cell extracts from wild-type strain. MDS1 duplex with labelling on 5' end of each constitutive oligonucleotide was incubated in the presence wild-type yeast whole cell extract for the indicated times and then processed as in Materials and Methods.
